# Severe traumatic brain injury- physician provided pre-hospital care and early neurosurgical treatment are associated with improved survival

**DOI:** 10.1186/1757-7241-23-S2-O9

**Published:** 2015-09-11

**Authors:** Toni Pakkanen, Ilkka Virkkunen, Antti Kämäräinen, Tom Silfvast, Tarja Randell, Heini Huhtala, Arvi Yli-Hankala

**Affiliations:** 1 Department of Anaesthesia, Tampere University Hospital, Tampere, Finland; 2 Tays Emergency Medical Service, Tampere University Hospital, Tampere, Finland; 3 Department of Anaesthesia and Intensive Care, Helsinki University Hospital, University of Helsinki, Helsinki, Finland; 4 School of Health Sciences, University of Tampere, Tampere, Finland; 5 Medical School, University of Tampere, Tampere, Finland

## Background

Worldwide, traumatic brain injury (TBI) is a leading cause of death and permanent disability[1]. Early and appropriate management of TBI is critical to the survival of these patients [1]. The aim of this study was to compare the outcome of TBI patients in two emergency medical service (EMS) systems.

## Method

A 6-year period observational data on pre-hospital TBI management in physician versus paramedic staffed EMS systems were retrospectively analysed. Inclusion criteria were isolated TBI with Glasgow coma scale (GCS) ≤ 8 on-scene or during transportation. Patients with life-threatening multiple trauma, secondary transfers and patients deceased on-scene were excluded. Evaluation was based on patient records one year after the incident. For assessment of neurological outcome, modified Glasgow Outcome Score (GOS) was used. The time and cause of death were recorded.

## Results

The physician (n = 275) and paramedic (n = 183) EMS patient groups were similar regarding demographic variables, mechanism of injury, time to reach the patient and first recorded on-scene GCS. Airway was secured in physician EMS group in 98 % and paramedic EMS group in 16 % of the patients (p < 0.001). Emergency neurosurgery was performed on 45 % and 30 % of the patients after hospital admission (p < 0.001). A statistically non-significant trend towards better neurological outcome was observed favouring physician provided pre-hospital care - 38 % of the physician and 31 % of the paramedic treated EMS patients had a good neurological recovery (GOS 4-5) with independent life one year after the event. Correspondingly, the overall one-year mortality rate was lower in the physician staffed EMS group: 43 % vs. 57 % (p < 0.01).

## Conclusion

TBI patient mortality was significantly lower and good neurological outcome higher in patients treated by the physician EMS group compared to the paramedic EMS group.
